# *ab initio* Energetics and Thermoelectric Profiles of Gallium Pnictide Polytypes

**DOI:** 10.1038/s41598-019-41982-9

**Published:** 2019-04-10

**Authors:** Trupti K. Gajaria, Shweta D. Dabhi, Prafulla K. Jha

**Affiliations:** 1 Department of Physics, Faculty of Science, The Maharaja Sayajirao University of Baroda, Vadodara, 390002 Gujarat India; 2 P. D. Patel Institute of Applied Science, Charotar University of Science and Technology, CHARUSAT campus, Changa, 388421 Gujarat India

## Abstract

The *state-of-the-art* Density Functional Theory (DFT) is utilized to investigate the structural, electronic, vibrational, thermal and thermoelectric properties of gallium pnictides GaX (X = P, As, Sb) in cubic zincblende (ZB) and hexagonal wurtzite (WZ) phases. The lattice parameters, bulk modulus, energy band nature and bandgap values, phonon, thermal and thermoelectric properties are revisited for ZB phase while for WZ phase they are predictive. Our results agree reasonably well with the experimental and theoretical data wherever they are available. The phonon dispersion curves are computed to validate the dynamic stability of these two polytypes and for further investigating the thermal and thermoelectric properties. Our computed thermoelectric figure of merit ZT gives consistent results with highest observed magnitude *of 0.72 and 0.56* for GaSb compound in ZB and WZ phases respectively. The first time calculated temperature variation of lattice thermal conductivity for WZ phase shows lower value than ZB phase and hence an important factor to enhance the figure of merit of considered gallium pnictides in WZ phase. Present results validate the importance of GaX in high temperature thermoelectric applications as the figure of merit ZT shows enhancement with significant reduction in thermal conductivity at higher temperature values.

## Introduction

Necessity to overcome the energy crises and desire to fulfill the energy demands of mankind, the researchers have been immensely studying various III-V compounds for developing green energy harvesting and storage devices^1–3^. This class of materials contribute to many cutting-edge technologies such as green energy harvesting photovoltaic (PV)^4–6^, nanoelectronics^7^, thermoelectric^8,9^, optoelectronic^10^, sensors^11^, visible/IR emitters^12,13^, and hybrid complementary metal oxide semiconductors (CMOS)^14^ due to their moderate and direct energy gap, good mechanical strength and structural polytypic behavior. Three eminent Japanese scientists, Nakamura, Amano and Akasaki shared the Nobel prize in Physics-2014 and set up a benchmark for III-V group compounds through their groundbreaking novel research on gallium nitride (GaN) based heterostructures which led to the discovery of blue light emitting diode (blue-LED)^12^. One of the recent studies reported by Lumb *et al*.^15^ demonstrates 44.5% efficient gallium antimonide (GaSb) based solar cells which are capable of absorbing IR photons from the sunlight. The revolutionary contributions of III-V compounds in major areas of technology have brought them in recent focus and their further investigations may open up new perspectives in developing suitable materials for device technologies. However, this demands a rigorous investigation to understand the potential outcomes of these compounds in different phases in which they exist under different conditions^16,17^. The zincblende (ZB) and wurtzite (WZ) are the two extensively studied III-V polytypes for their mechanical, electronic, optoelectronic and vibrational properties in bulk and nano regimes during the last few years^18–41^.

Recently, the thermoelectric studies on variety of semiconductors including III-V compounds^8,9,42–50^ have gained greater attention due to the importance of thermal management in devices especially when, the device sizes are continuously decreasing. The literature reveals many theoretical^21–25,30,39^ and experimental^18–20,26–29,31,32,36,40,41^ studies on the structural, mechanical, electronic and vibrational properties of gallium pnictides; (GaX; X = P, As, Sb). However, a lack of systematic study on thermoelectricity in GaX polytypes is observed. While there exist few scattered studies on lattice conductivity computations in ZB phase of bulk GaX, same is completely missing for WZ phase^51–53^.

The thermoelectric efficiency of a material can be described by the parameters, electronic figure of merit ZT_e_ and overall figure of merit (electron + phonon) ZT, defined as

1 $${{\rm{ZT}}}_{{\rm{e}}}=\frac{{{\rm{S}}}^{2}{\rm{\sigma }}\,{\rm{T}}}{{{\rm{\kappa }}}_{{\rm{e}}}}$$

2 $${\rm{ZT}}=\frac{{{\rm{S}}}^{2}{\rm{\sigma }}\,{\rm{T}}}{{{\rm{\kappa }}}_{{\rm{e}}}+{{\rm{\kappa }}}_{{\rm{l}}}}$$

where, S is Seebeck co-efficient, σ is electrical conductivity, T is temperature and κ_e_ and κ_l_ are the electronic and lattice contributions to the thermal conductivity respectively. As it can be seen from the equation (2), apart from electronic contributions, the thermoelectric figure of merit ZT is very sensitive to the thermopower also known as Seebeck co-efficient S and lattice thermal conductivity κ_l_. Reduction in κ_l_ and enhancement in S significantly improves ZT, but as these parameters are coupled with each other and their dependence on the crystal structure and carrier concentration increases the complexity^54^. Literature survey reveals many theoretical reports on lattice conductivity computation for III-V compounds and alloys taking into consideration various aspects/parameters affecting it^8,44,46,51–53^. Liu *et al*.^44^ have demonstrated thermoelectric behavior of WZ bulk GaN and Al doped GaN and found that thermoelectric figure of merit ZT for Al based GaN alloy attains optimum value of 0.2 at 1000 K. Bahk *et al*.^46^ computed thermoelectric parameters of III-V quaternary alloy and reported the value of ZT that can reach up to 1.3 at 1000 K. A very systematic first principles based DFT study of isotope effect on lattice conductivity is presented by Lindsay *et al*.^51^ for GaN and related III-V compounds. The authors report 65% increase in κ_l_ at room temperature subjected to the isotopic enrichment effect. The recent theoretical report by Luo *et al*.^52^ elaborates phonon mode dependent contributions to lattice thermal conductivity in GaAs and suggests 90% contribution in lattice conductivity arising from acoustic phonon modes. The room temperature thermal conductivity of group-IV and III-V semiconductor alloys was analyzed by Adachi^53^ using a simplified model based on alloy scattering effects. Further, theoretical calculations of thermoelectric properties of III-V nanowires as a function of nanowire thickness was studied by Mingo^8^ that suggests ZT~6 can be achieved in case of InSb nanowires validating it to be a suitable candidate for thermoelectric applications.

The signature contributions of III-V compounds in various cutting-edge technologies and urge to get more insight to their phase dependent properties, we have focused this study on the two polytypic phases; zincblende (ZB) and wurtzite (WZ), of gallium pnictides GaX (X = P, As, Sb) for investigating the structural, electronic, vibrational, thermal and thermoelectric properties. The prime motivation of the present study is to add up the missing data on GaX compound polytypes in the database and set up a clear link between the properties of two polytypes by providing a substantial insight into the phase dependent thermoelectric mechanism. To validate the consistency of our *ab initio* calculations and support our results, we are revisiting the electronic and vibrational properties of GaX compounds for both phases.

## Results and Discussion

### Morphology Optimization

The zincblende and wurtzite are both close packed crystal structures with slightly different atomic arrangements arising due to different stacking layers. The atomic arrangement in cubic ZB is of …BABABAB… type with basis atoms while hexagonal WZ has the stacking type …CABCABCAB… The cubic ZB which belongs to ${\rm{F}}\bar{4}3m\,$space group (no. 216) has in total two atomic contributions per unit cell while the hexagonal WZ belonging to P6_3_mc space group (no. 186) has four atomic contributions per unit cell. The stacking mechanism is responsible for the difference in geometry and, the material properties which needsto be addressed, as the properties of the nano devices are critically phase/morphology dependent. Subject to the above fact, we first optimized the crystal geometries of GaX for ZB and WZ phases by minimizing the total energy self-consistently (see Methods Section). The lattice constants computed for GaP, GaAs and GaSb in ZB and WZ phases presented in Table 1 are slightly underestimated (1.2–2%) compared to the reported experimental values^18–20^. This can be attributed to the local density approximation to exchange correlation which usually over binds the atoms by 1–2%^55^, though the c/a ratio for WZ compounds is in good agreement with ideal $\sqrt{8/3\,}$ value with slight deviation of ~0.9%. The bulk modulus and its pressure derivative for GaX compounds in ZB and WZ phases presented in Table 1 are computed by performing energy-volume calculations followed by a fitting using Birch-Murnaghan equation of state^56^. The bulk modulus of GaX compounds are in good agreement with the respective reported experimental values^18^. A critical analysis of Table 1 clearly shows that the lattice parameters and the bulk modulus increase and decrease respectively, going from GaP to GaSb in both phases; i.e. as anion mass increases, its radius causes an increase in lattice constant and a reduction in the bulk modulus. Table 1 also validates our calculations, as it shows good agreement with prior local density approximation(LDA) based reports^21,22,25^.

**Table 1:** The lattice parameters, bulk modulus and pressure derivative of bulk modulus of GaX compounds in ZB and WZ phases.

System	Property		Lattice parameter (Å)		Bulk modulus (GPa)		Pressure derivative of bulk modulus	
	Phase	ZB	WZ		ZB	WZ	ZB	WZ
	Parameter	a	A	c/a	B_0_		${{\bf{B}}}_{{\bf{0}}}^{{\boldsymbol{^{\prime} }}}$	
GaP	Present	5.338	3.762	1.649	91.5	88.8	4.40	4.41
	**Exp**.	**5.451** ^**a**^	**3.842** ^**b**^	**1.649** ^**c**^	**88** ^**a**^	—	—	—
	Other	5.332^d^	3.763^d^	1.639^d^	92.1^d^	91.23^d^	4.339^d^	4.3437^d^
		5.43^e^	3.800^k^	1.650^k^	91.9^e^		4.58^e^	
		5.41^f^			91.5^f^		4.50^f^	
		5.54^g^			79.1^g^		4.45^g^	
		5.52^h^			77.3^h^		4.52^h^	
		5.501^i^			77.21^i^		4.88^i^	
		5.451^j^						
		5.397^k^						
GaAs	Present	5.547	3.909	1.649	75.4	74.1	4.52	4.53
	**Exp**.	**5.649** ^**a**^	**4.021** ^**c**^	—	77^a^	—	—	—
	Other	5.530^d^	3.912^d^	1.637^d^	75.7^d^	74.73^d^	4.487^d^	4.5048^d^
		5.63^e^	3.953^k^	1.650^k^	77.1^e^		4.30^e^	
		5.61^f^			76.0^f^		4.33^f^	
		5.77^g^			64.4^g^		4.86^g^	
		5.75^h^			60.2^h^		5.20^h^	
		5.733^i^			60.83^i^		4.60^i^	
		5.653^j^						
		5.440^k^						
GaSb	Present	6.005	4.232	1.648	56	55.4	4.64	4.64
	**Exp**.	**6.081** ^**a**^	—	—	**56** ^**a**^	—	—	—
	Other	5.981^d^	4.234^d^	1.635^d^	56.7^d^	55.80^d^	4.662^d^	4.6695^d^
		6.08^e^	4.233^k^	1.653^k^	60.0^e^		4.78^e^	
		6.06^f^			56.6^f^		4.80^f^	
		6.24^g^			49.1^g^		4.66^g^	
		6.22^h^			45.9^h^		4.16^h^	
		6.193^i^			45.92^i^		5.16^i^	
		6.118^j^						
		6.018^k^						

^a^ref.^18^ [Exp],

^b^ref.^19^ [Exp],

^c^ref.^20^ [Exp],

^d^ref.^21^ [LDA],

^e–h^ref.^22^ [LDA and GGA],

^i^ref.^23^ [GGA]

^j^ref.^24^ [Empirical Method] and

^k^ref.^25^ [LDA].

### Electronic Transport

**Figure 1:**
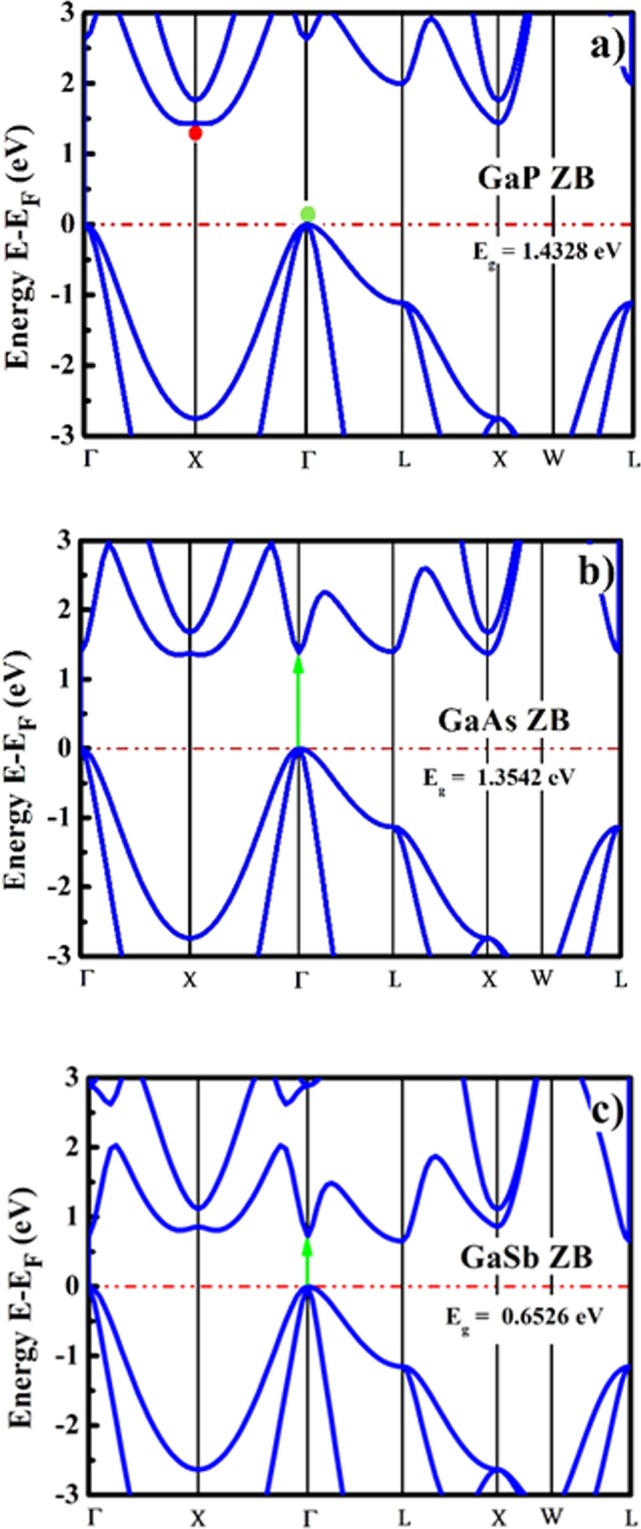
Electronic band structures of GaX compounds in ZB phase.

**Figure 2:**
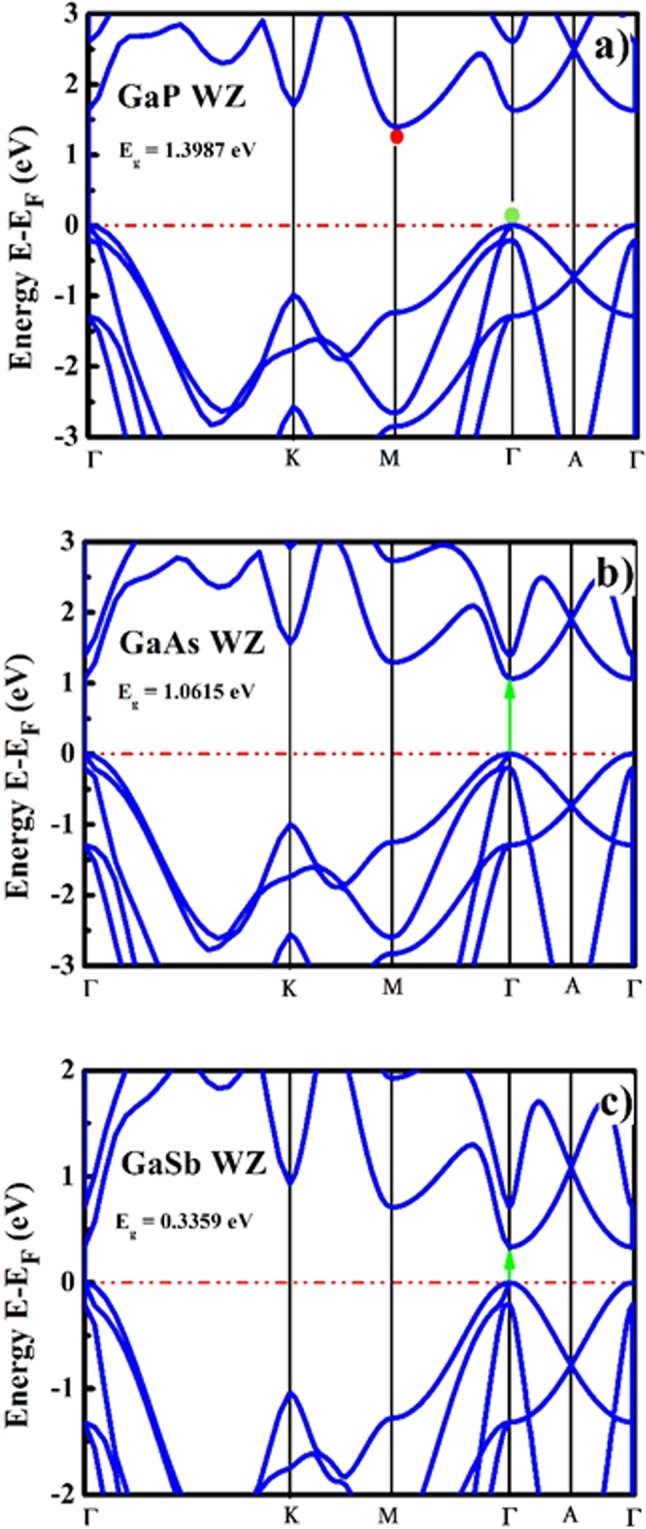
Electronic band structures of GaX compounds in WZ phase.

The electronic properties such as electronic band structure, conduction band minima (CBM) and valence band maxima (VBM) energies, electronic density of states (DOS) and partial density of states (PDOS) were obtained using electronic band structure calculations along the high symmetry k-path of the irreducible Brillouin zone. The computed and reported electronic band parameters are enlisted in Table 2. The electronic band structures for ZB and WZ GaX compounds are presented in Figs 1 and 2, which show that the bandgaps reduce for both phases as going from GaP to GaSb attributed to increase in anion mass and reduction in lattice parameters. The computed energy band gaps for ZB compounds are comparable with the reported experimental data for bulk GaX compounds with slight underestimation of the magnitudes^26–29^. The standard DFT usually underestimates the value of bandgap due to the effect of self-interaction^57^ and the derivative discontinuity^58^ yet, it gives better results for III-V compounds than generalized gradient approximation (GGA)^55^. The literature reveals similar electronic dispersion using both hybrid and LDA but a lower bandgap by LDA than hybrid functionals^59^. Furthermore, second important reason for using LDA in the present calculations is, not having hybrid functionals implemented for thermoelectric transport calculations which is one of the major aims of the present study. The nature of the bandgap which can be confirmed by locating the VBM and CBM energies in the electronic band structures is direct in case of GaAs and GaSb in both phases while it is found to be indirect for GaP in both phases.

**Table 2:** The electronic bandgaps and bandgap types of GaX compounds in ZB and WZ phases.

System	Energy gap (eV)					Bandgap Type	
		ZB			WZ	ZB	WZ
		${{\bf{E}}}_{{\bf{g}}}^{{\boldsymbol{\Gamma }}-{\boldsymbol{\Gamma }}}$	${{\bf{E}}}_{{\bf{g}}}^{{\boldsymbol{\Gamma }}-{\bf{X}}}$	${{\bf{E}}}_{{\bf{g}}}^{{\boldsymbol{\Gamma }}-{\bf{L}}}$	${{\bf{E}}}_{{\bf{g}}}^{{\boldsymbol{\Gamma }}-{\boldsymbol{\Gamma }}}$		
GaP	Present	2.632	1.4328	1.9652	1.5895(1.3987)	Indirect	Indirect
	**Exp**.	**2.78** ^**a**^	**2.26** ^**a**^	**2.6** ^**a**^	**2.09** ^**k**^		
	Other	2.29^e^	2.55^e^	3.25^e^	1.4389^g^		
		1.46^f^	1.68^f^	1.51^f^	2.251^j^		
		2.438^g^	—	—			
		2.0^h^	2.50^h^	2.09^h^			
		2.774^i^	2.25^i^	2.6^i^			
		2.47^m^					
		2.85^m^					
GaAs	Present	1.3542	1.3391	1.3391	1.0615	Direct	Direct
	**Exp**.	**1.42** ^**b**^	**1.81** ^**b**^	**1.72** ^**b**^	**1.459** ^**l**^		
	Other	1.21^e^	2.32^e^	1.65^e^	0.7781^g^		
		0.05^f^	1.49^f^	0.79^f^	1.503^j^		
		1.008^g^	—	—			
		0.49^h^	2.40^h^	1.3^h^			
		1.42^i^	1.81^i^	1.72^i^			
		1.21^m^					
		1.44^m^					
GaSb	Present	0.678	0.8156	0.6086	0.3359	Direct	Direct
	**Exp**.	**0.725** ^**c**^	**1.03** ^**d**^	**0.761** ^**d**^	—		
	Other	1.00^e^	1.46^e^	1.13^e^	0.1673^g^		
		−0.35^f^	0.94^f^	0.28^f^	0.509^j^		
		0.547^g^	—	—			
		0.4^h^	1.60^h^	0.8^h^			
		0.715^i^	1.012^i^	0.777^i^			
		0.72^m^					
		0.81^m^					

^a^Ref.^26^ [Exp]

^b^Ref.^27^ [Exp],

^c^ref.^28^ [Exp],

^d^ref.^29^ [Exp],

^e-f^ref.^22^ [LDA and GGA],

^g^ref.^21^ [LDA],

^h^ref.^23^ [GGA],

^i^ref.^24^ [Empirical Method],

^j^ref.^30^ [Empirical Method],

^k^ref.^31^ [Exp],

^l^ref.^32^ [Exp] and

^m^ref.^59^ [Hybrid Functional].

**Figure 3:**
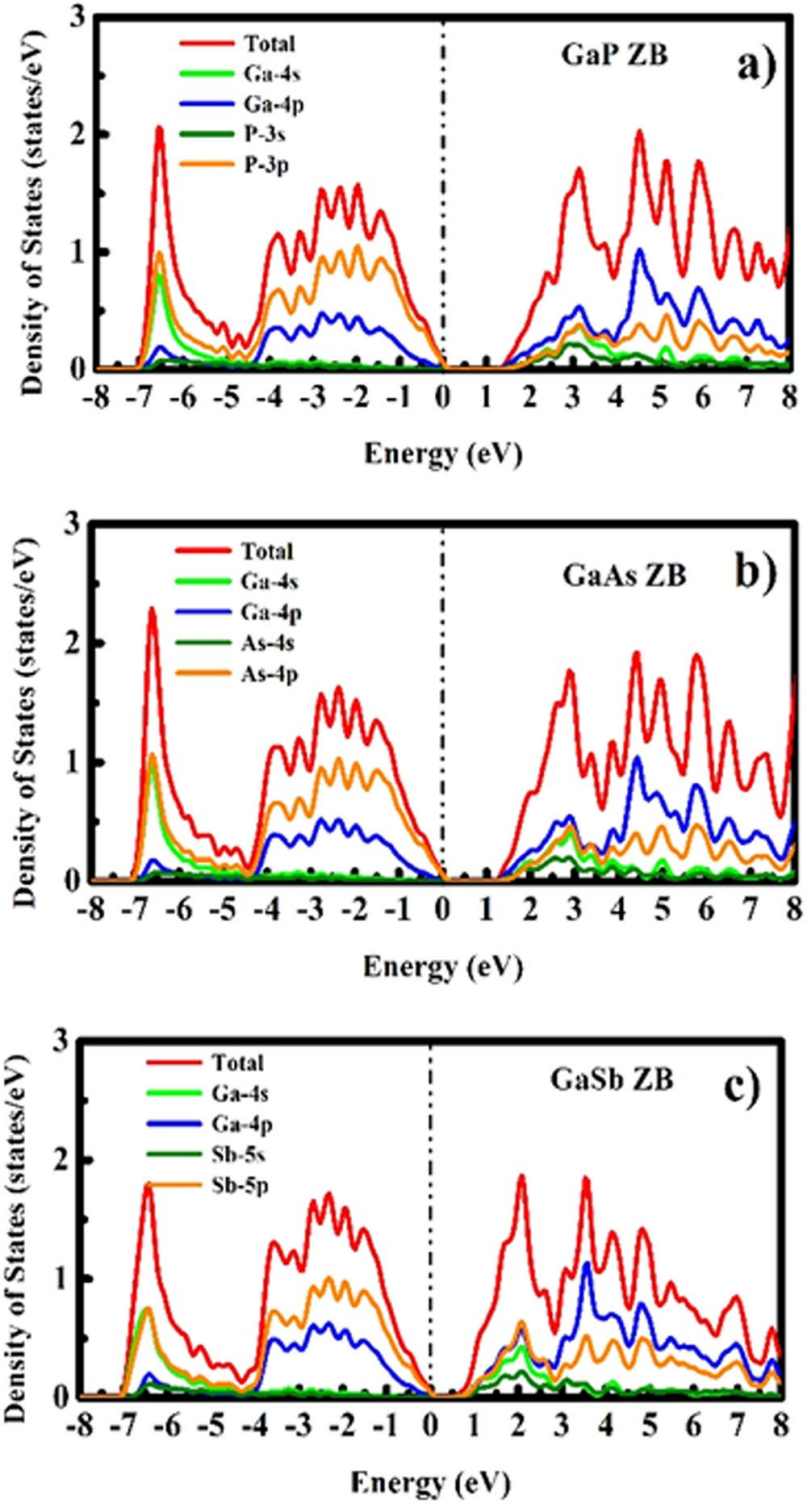
Electronic density of states of GaX compounds in ZB phase.

**Figure 4:**
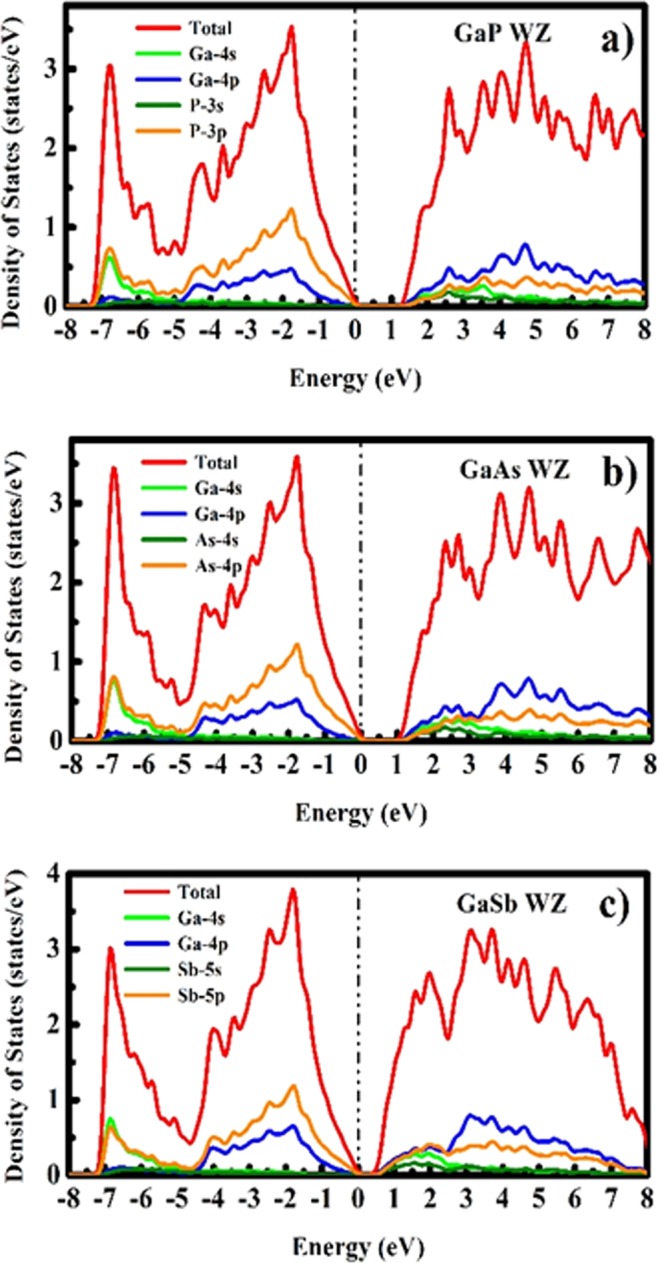
Electronic density of states of GaX compounds in WZ phase.

As there does not exist any experimental data for bulk GaX compounds in WZ phase, we compared our computed band gap values with the experimental data reported for WZ GaX nanowires (NWs). The photoluminescence (PL) study was performed on WZ GaP NWs and a direct bandgap of 2.09 eV^31^ was measured whereas, we observe an indirect nature of the bandgap (${{\rm{E}}}_{{\rm{g}}}^{{\rm{\Gamma }}-{\rm{M}}}$) with a magnitude of 1.3987 eV (${{\rm{E}}}_{{\rm{g}}}^{{\rm{\Gamma }}-{\rm{\Gamma }}}=1.5895\,{\rm{eV}}$). For WZ GaAs, the resonant Raman study was performed on WZ GaAs NW and an optical bandgap of 1.459 eV was measured^32^, which is comparable with our computed direct bandgap of 1.0615 eV. The predicted band gap nature of WZ GaSb is direct with the magnitude of 0.3359 eV, which has a deviation from the prior LDA and pseudopotential based reports^21,30^ which can be attributed to the overestimation of the computed c/a ratio (c/a = 1.648). To understand the specific orbital contribution to the electronic properties and underlying orbital hybridization of GaX compounds, we have computed the total and partial electronic density of states (DOS and PDOS) for both phases of GaX compounds. The PDOS plot depicted in Figs 3 and 4 reveals that the Ga *4p-orbital* having only one electron in ZB and WZ phase contributes more to conduction band energies and is thus responsible for electronic conduction whereas, P, As and Sb *p-orbitals* having three electrons contribute more to valence band. The *s-orbital* electrons almost contribute equally higher to valence band energies for the particular system.

### Phase Dependent Frequency Analysis

To analyze the morphology dependent vibrational behavior and to validate the dynamic stability of the considered systems, we have computed the phonon dispersion curves (PDCs) and phonon density of states (PHDOS) which give peculiar insight to the dynamic stability of the crystal structure and spatial distribution of the vibrational modes. In addition, phonon calculations can also be utilized for computing thermodynamic functions and thermoelectric properties with the parameters affecting them which are notreported for GaX compounds so far. As ZB and WZ are polytypes, it is necessary to analyze their vibrational behavior to understand the difference in their vibrational energies and their contribution to allied properties. To investigate the dynamic stability of the proposed system and for further analysis of the thermal and thermoelectric properties, the phonon dispersion curves (PDCs) along the high symmetry points of irreducible Brillouin zone boundaries were computed using linear response theory based DFPT approach^60^ which directly computes second order derivative of energy and generates second order interatomic harmonic force constants (IFCs).

**Figure 5:**
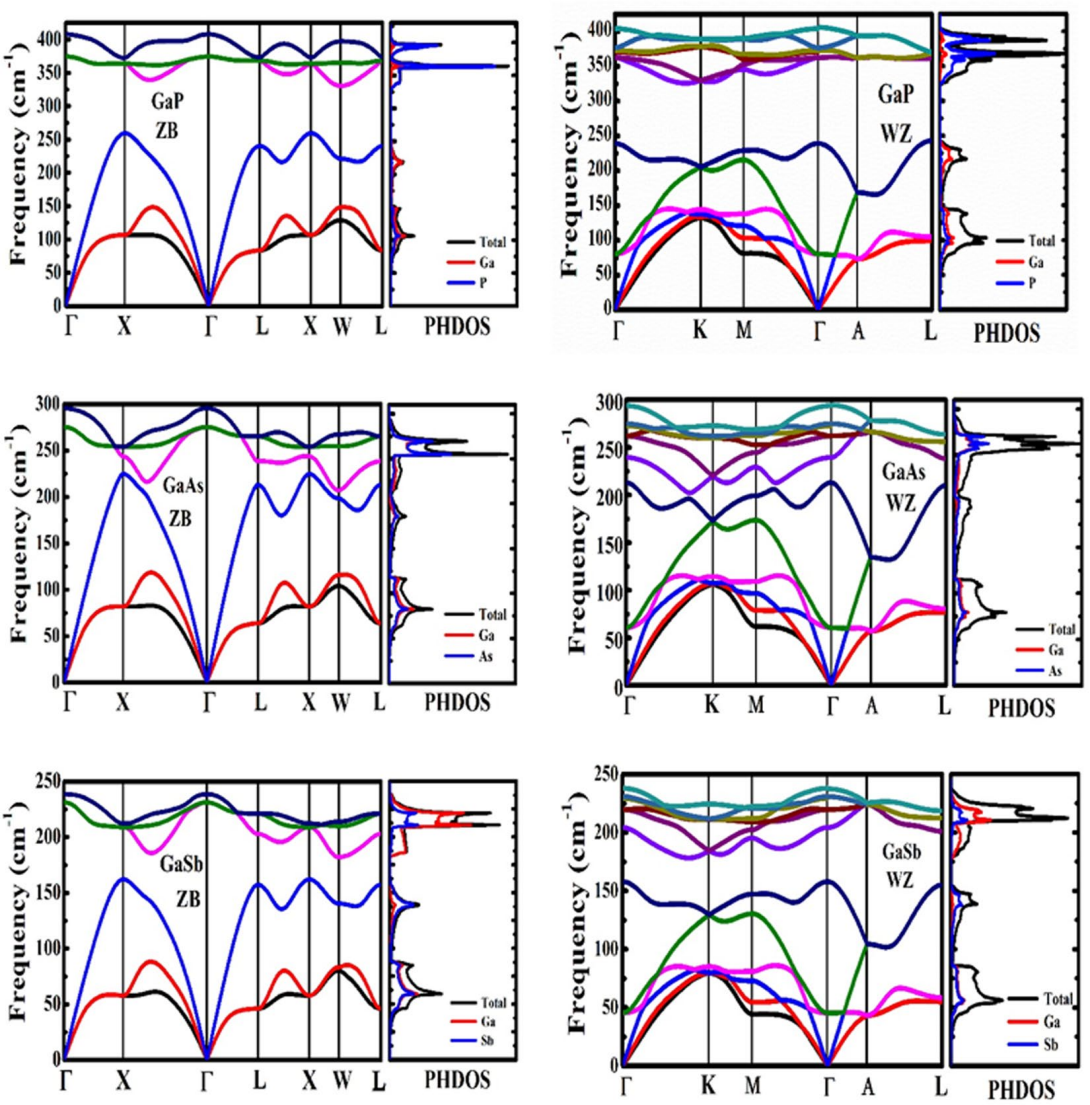
Phonon dispersion curves along with the phonon density of states (PHDOS) in ZB and WZ phases.

The vibrational energies in the PDCs are classified in two categories: acoustic and optic phonon modes which are subjected to the direction of the mechanical vibrations of the system atoms. The acoustic modes in the vibrational spectra result from the mechanical vibrations of the nearest atoms in the same phase whereas the optical modes result due to opposite or out of phase vibrational motions of the system atoms. As the system considered here is made up of two different atomic species, the mechanical vibrations and their corresponding frequencies are quite different which give rise to non-degeneracy between the longitudinal optic (LO) and transverse optic (TO) modes known as LO-TO splitting. As the number of atoms per unit cell is two for ZB and four for WZ, the PDCs for GaX compounds in ZB phase have a total of six phonon branches; three acoustic and three optical whereas, in WZ phase, the PDCs have a total of twelve phonon branches; three acoustic, three lower lying optical branches and six higher optical branches. The acoustic phonons are classified as one longitudinal acoustic (LA) and two transverse acoustic (TA) modes. All these three acoustic modes are triply degenerate at Γ point-the center of the Brillouin zone. The computed PDCs are presented in Fig. 5 which reveals that the highest observed optical frequencies show significant diminution approaching from GaP to GaSb in both phases. The LO-TO splitting which is merely due to dipole-dipole contribution shows decrease in magnitude while moving from GaP to GaAs to GaSb for both phases. The decrease in the vibrational frequencies is subjected to the increase in the anion mass of the system. The computed vibrational frequencies throughout the Brillouin zone do not show any imaginary component, validating the dynamic stability of the system in both phases.

The phonon frequencies calculated at high symmetry points (Γ, X and L) for ZB GaX compounds together with the available experimental data are presented in Table 3. As can be seen from the data presented in Table 3, the computed phonon frequencies are in excellent agreement with the reported experimental^33–38^ and theoretical^25,39^ data, except for GaP for which some of the frequencies are overestimated due to 2% underestimation of lattice parameter from experimental data^18^. As the ZB and WZ are two polytypic phases and differ only due to difference in stacking layers, we can predict the phonon dispersion of WZ structure for [0001] direction by folding the corresponding ZB phonon dispersion curve along the [111] direction^61^. As the number of atoms in WZ unit cell is double than that of ZB, and the c/a ratio computed for WZ polytypes slightly differ from the ideal value $\sqrt{8/3}$, the WZ PDCs show difference in phonon frequencies and as a consequence, new phonon modes at Γ point of the BZ are introduced^61^. According to the group theory, the WZ polytypic structure gives rise to eight phonon modes at Γ point. These include 2A_1_, 2B_1_, 2E_1_ and 2E_2_ modes out of which the B_1_ modes are silent modes. The non-polar ${{\rm{E}}}_{2}^{{\rm{h}}}$ mode, the characteristic phonon mode for WZ phase, is located at 368, 268 and 227 cm^−1^ for GaP, GaAs and GaSb respectively. For GaP, our predicted frequency of 362 cm^−1^ corresponds to degenerate E1(TO) and A1(TO) modes that precisely match with the experimentally observed frequency of 361 cm^−1^ for both modes^40^, though the prior LDA based first principles calculation shows disagreement with the experimentally observed frequency exhibiting a markable gap of 5 cm^−1^ between these two modes^25^. The frequencies of other phonon modes are slightly deviated from the experimental values; whereas for GaAs, we observed excellent agreement with the reported experimental data^41^. There is no experimental data on vibrational profile of GaSb in WZ phase and so we compared our results with prior DFT based data^25^ and found good agreement between both of them. The slight deviation in phonon frequencies of GaX compounds is subjected to exclusion of *d-orbital* electrons in the pseudopotentials of the respective elements. The exclusion of the *d-orbital* electrons reduces screening effects, thus overestimating the bonding strength^61^.

**Table 3:** The acoustic and optic phonon frequencies at high symmetry points of BZ and the Born effective charges (Z^*^) and high frequency di-electric constants (ε) for ZB GaX compounds.

System		Γ_TO_	Γ_LO_	X_TA_	X_LA_	X_TO_	X_LO_	L_TA_	L_LA_	L_TO_	L_LO_	Z^*^	ε
GaP	Calc.	371	405	103	257	363	372	80	236	368	373	2.06	9.80
	**Exp** ^**a,b,e**^	**366**	**404**	**103**	**250**	**379**	**358**	**64**	**212**	**353**	**368**	**2.04**	**9.11** ^**g**^
	Other^c^	364	394	93	247	361	366	68	228	362	370	2.06	8.93
GaAs	Calc.	275	295	81	224	243	253	63	212	238	265	2.03	11.50
	**Exp** ^**d,e**^	**271**	**293**	**82**	**225**	**257**	**240**	**63**	**207**	**264**	**242**	**2.07**	**10.9** ^**g**^
	Other^c,h^	270	286	85	220	256	243	70	209	265	240	2.08	10.83
		271	291	82	223	254	240	63	210	263	238	2.07	12.3
GaSb	Calc.	230	238	57	162	209	212	46	157	203	221	1.62	15.89
	**Exp** ^**f**^	**224**	**233**	**57**	**166**	**212**	**212**	**46**	**153**	**205**	**216**	**2.15**	**14.4**
	Other^c,h^	228	234	64	161	213	214	53	157	223	207	1.61	12.87
		230	237	57	162	210	211	45	157	203	221	1.73	18.1

^a^Ref.^33^ [Exp],

^b^ref.^34^ [Exp],

^c^ref.^25^ [LDA],

^d^ref.^35^ [Exp],

^e^ref.^36^ [Exp],

^f^ref.^37^ [Exp],

^g^ref.^38^ [Exp] and

^h^ref.^39^.

The Born effective charges Z^*^ and high frequency di-electric constantsε for ZB and WZ phases of GaX compounds are listed in Tables 3 and 4 respectively. We can quantify the coupling arising due to interaction of the optic phonons and the electric field by the Born effective charge Z^*^, which is found to be isotropic for cubic ZB phase as ${{\rm{Z}}}_{{\rm{xx}}}^{\ast }={{\rm{Z}}}_{{\rm{yy}}}^{\ast }={{\rm{Z}}}_{{\rm{zz}}}^{\ast }$, whereas the same shows anisotropic nature for WZ phase as ${{\rm{Z}}}_{{\rm{xx}}}^{\ast }={{\rm{Z}}}_{{\rm{yy}}}^{\ast }\ne {{\rm{Z}}}_{{\rm{zz}}}^{\ast }$. We therefore enlist the average Born effective charges for both the phases in Tables 3 and 4, which show excellent agreement with the reported experimental data^41^ for ZB phase. The average values of high frequency di-electric constants show mass dependent trend in both phases, as it increases with increase in anion mass. There is no experimental data available to compare with the WZ phase. Further, for ZB phase, we found ε to be overestimated from the reported experimental data^38^. The reason for the overestimation is explained elsewhere and is in agreement with the observed trend^61^.

**Table 4:** The vibrational frequencies of phonon modes, Born effective charges (Z^*^) and high frequency di-electric constants (ε) of WZ GaX compounds.

System	Zone Center Phonon frequency (cm^−1^)									Effective Charge	Di-electric Constant
		${{\bf{E}}}_{{\bf{2}}}^{{\bf{1}}}$	${{\bf{B}}}_{{\bf{1}}}^{{\bf{1}}}$	A_1_(TO)	E_1_(TO)	${{\bf{E}}}_{{\bf{2}}}^{{\bf{h}}}$	${{\bf{B}}}_{{\bf{1}}}^{{\bf{h}}}$	A1(LO)	E1(LO)	Z^*^	ε
GaP	Calc.	78	236	362	362	368	375	407	402	2.019	9.75
	Exp^b^	80	215	361	361	353	383	391	397	—	—
	Other^a^	76	232	361	366	358	375	413	393	2.64	8.93
GaAs	Calc.	59	211	239	262	261	274	293	294	1.98	11.14
	Exp^c^	59	206	—	267	259	234	291	—	—	—
	Other^a^	59	207	267	271	261	239	287.5	287	2.01	10.82
GaSb	Calc.	43	156	219	202	227	229	239	236	1.57	14.67
	Other^a^	39	157	226	228	220	204	233	234	1.55	12.87

^**a**^Ref.^25^ [LDA]

^**b**^ref.^40^ [Exp]

^**c**^ref.^41^ [Exp].

Further, the partial phonon density of states (PHDOS) which requires the calculation of phonon modes in entire BZ, a real test of any approach^60,62^, is therefore computed to understand the contribution of individual atomic vibrations in acoustic and optical phonon modes. The PHDOS plots for GaX in both phases are shown in Fig. 5 along with the corresponding PDCs. In the case of GaP, the gallium (Ga) atoms contribute to acoustic and lower lying optical modes but the higher optical vibrational modes are strongly dominated by phosphorous atoms. For GaAs, there is no significant amount of variation in the masses of gallium and arsenic atoms hence, their contribution to acoustic and optical vibrational modes is almost equal in ZB and WZ phases which can be noticed by the overlapping of phonon densities in the PHDOS plots. However, for GaSb, the Ga and Sb atoms contribute equally in acoustic modes and the optic modes show intense contribution of Ga atoms in both phases.

The excellent agreement in phonon frequencies for GaAs and GaSb with the reported values validates the consistency of our calculation and provides impetus for further investigation of the system for proposed properties.

### Thermal and Thermoelectric Transport

**Figure 6:**
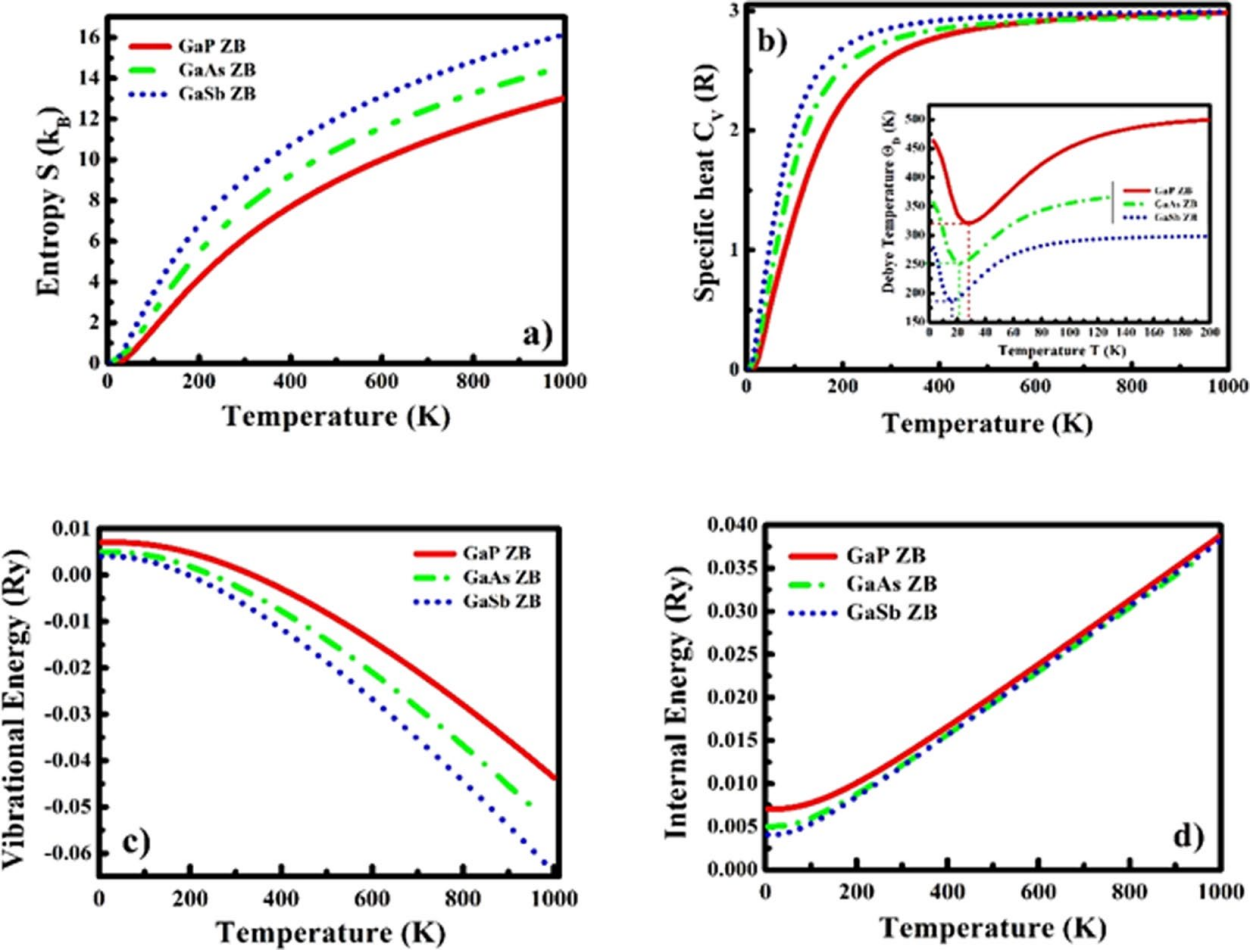
Thermodynamic functions (**a**) Entropy S (in units of k_B_), (**b**) specific heat C_ν_ (in units of R) (inset: Debye temperature Θ_D_), (**c**) Vibrational energy and (**d**) Internal energy as a function of temperature of GaX compounds in ZB phase.

**Figure 7:**
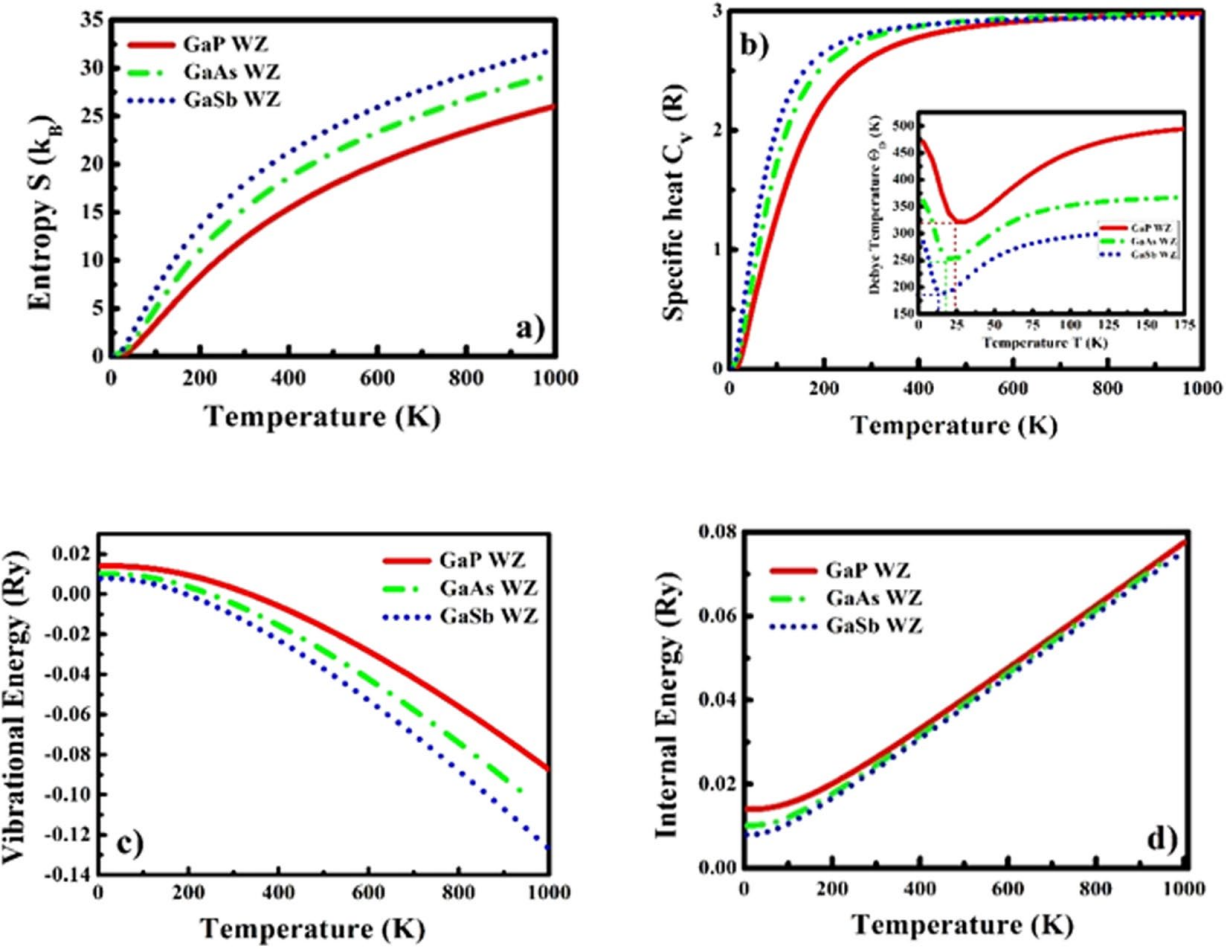
Thermodynamic functions (**a**) Entropy S (in units of k_B_), (**b**) specific heat C_ν_(in units of R)(inset: Debye temperature Θ_D_), (**c**) Vibrational energy and (**d**) Internal energy as a function of temperature of GaX compounds in WZ phase.

The thermodynamic functions such as lattice specific heat (C_ν_) at constant volume, Debye temperature (Θ_D_), entropy (S), internal and vibrational energies of GaX compounds in both phases are computed to investigate the thermal profiles of these compounds in both phases and validate their thermal stability. These volume dependent thermodynamic functions shown in Figs 6 and 7 for ZB and WZ phases respectively are computed based on the following equations under quasi-harmonic approximation (QHA)^63^.

3 $$E(T)={E}_{tot}+{E}_{zp}+\int \frac{\hslash \omega }{\exp (\frac{\hslash \omega }{kT})-1}F(\omega )d\omega $$

4 $$F(T)\,={E}_{tot}+{E}_{zp}+\,kT{\int }^{}F(\omega )\mathrm{ln}[1-\,\exp (-\frac{\hslash \omega }{kT})]d\omega $$

5 $$S(T)=k\,\{\int \frac{\hslash \omega }{\exp (\frac{\hslash \omega }{kT})-1}F(\omega )d\omega -\int (\omega )[1-\exp (-\frac{\hslash \omega }{kT})]d\omega \}$$

6 $$C{\rm{\nu }}(T)={\rm{k}}\frac{\int {(\frac{\hslash \omega }{kT})}^{2}exp(\frac{\hslash \omega }{kT})}{[\exp (\frac{\hslash \omega }{kT})-1]{}^{2}}F(\omega )d\omega $$

where, *E*_*tot*_ is total energy, *E*_*zp*_ is zero-point energy, $\hslash$ is reduced Plank’s constant and F(ω) represents the phonon density of states (PHDOS) computed under DFPT approach^60^.

As we can observe from Figs 6(a) and 7(a), the entropy (S) of the system increases as a function of temperature. The normalized lattice specific heat (C_υ_), a measure of energy carried by a phonon, is depicted in Figs 6(b) and 7(b), which approaches to 3 R (R = Universal Gas Constant) value at higher temperature validating the Dulong-Petit law. At lower temperatures, the C_v_ shows T^3^ dependence validating Debye’s law. The variation in Debye temperature (Θ_D_) with temperature can be clearly observed by the insets of Figs 6(b) and 7(b) as the trends of Θ_D_ for GaX compounds are almost similar for both phases and decrease with increase in anion mass. The internal (Figs 6(c) and 7(c)) and vibrational energies (Figs 6(d) and 7(d)) also follow similar trends for both phases and show mass dependency as, both energies become double in the case of WZ compounds due to twice number of atoms per unit cell in WZ phase compared to the ZB phase.

We now focus on our final objective of computing and understanding the thermoelectric transport properties of GaX compounds in ZB and WZ phases. The electronic and phonon contributions to thermoelectric transport were studied separately for getting clear insight to the role played by electrons and phonons to transport mechanism. (See Methods Section). The electronic contribution is evaluated by solving semi-classical Boltzmann transport equation (BTE) under constant relaxation time approach (CRTA) as implemented on BoltzTraP^64^. As a known fact, the CRTA based results strongly depend on the carrier relaxation time τ, we have computed, the relaxation time for each system and incorporated the same for accurately computing the electrical and electronic conductivities.

We first calculated carrier mobility µ (see Methods section), which was utilized for computing the relaxation time τ. It is a known fact that the magnitude of mobility μ decreases with increase in temperature T (see Supplementary Figs S2, S3) as a cause of increased ionic oscillations which further increases the rate of collisions, and as a result, the relaxation time τ decreases (see Supplementary Figs S2, S3). The computed parameters for evaluating hole mobility and the relaxation time for GaX compounds in ZB and WZ phases are presented in Table 5. Our computed room temperature values of relaxation time are 283 fs and 224 fs (for GaP), 293 and 200 fs (for GaAs), 312 and 181 fs (for GaSb) in ZB and WZ phases respectively. Our results for GaAs in ZB phase is in excellent agreement with the reported experimental value^65^. It can be observed that the magnitude of relaxation time increase with increase in anion mass for ZB phase; however for WZ phase, we surprisingly observed different trend showing decrease in relaxation time subjected to increase in anion mass. Further, it can also be observed that the magnitude of τ in WZ phase is lower than that of the ZB phase. The difference in the observed trend can be attributed to the anisotropic nature of WZ phase. Furthermore, the deformation potential E_1_ and elastic constant C_ii_ significantly affect the mobility and in turn the relaxation time.

**Table 5:** Calculated hole effective mass(m_h_^*^), deformation potential(E_1_), elastic constant(C_ii_), hole mobility(µ) and relaxation time(τ) of GaX compounds in ZB and WZ phases.

System	Phase	${{\boldsymbol{m}}}_{{\boldsymbol{h}}}^{\ast }$ (m_e_)	E_1_ (eV)	C_ii_ (GPa)	µ (m^2^/V.s)	τ (fs)
GaP	ZB	0.5	7.11	149.7	983	283
	WZ	0.62	7.47	180.3	628	224
GaAs	ZB	0.47	6.66	123	1090	293
	WZ	0.64	7.09	152.1	544	200
GaSb	ZB	0.39	6.6	91.4	1380	312
	WZ	0.58	7.1	118.5	543	181

**Figure 8:**
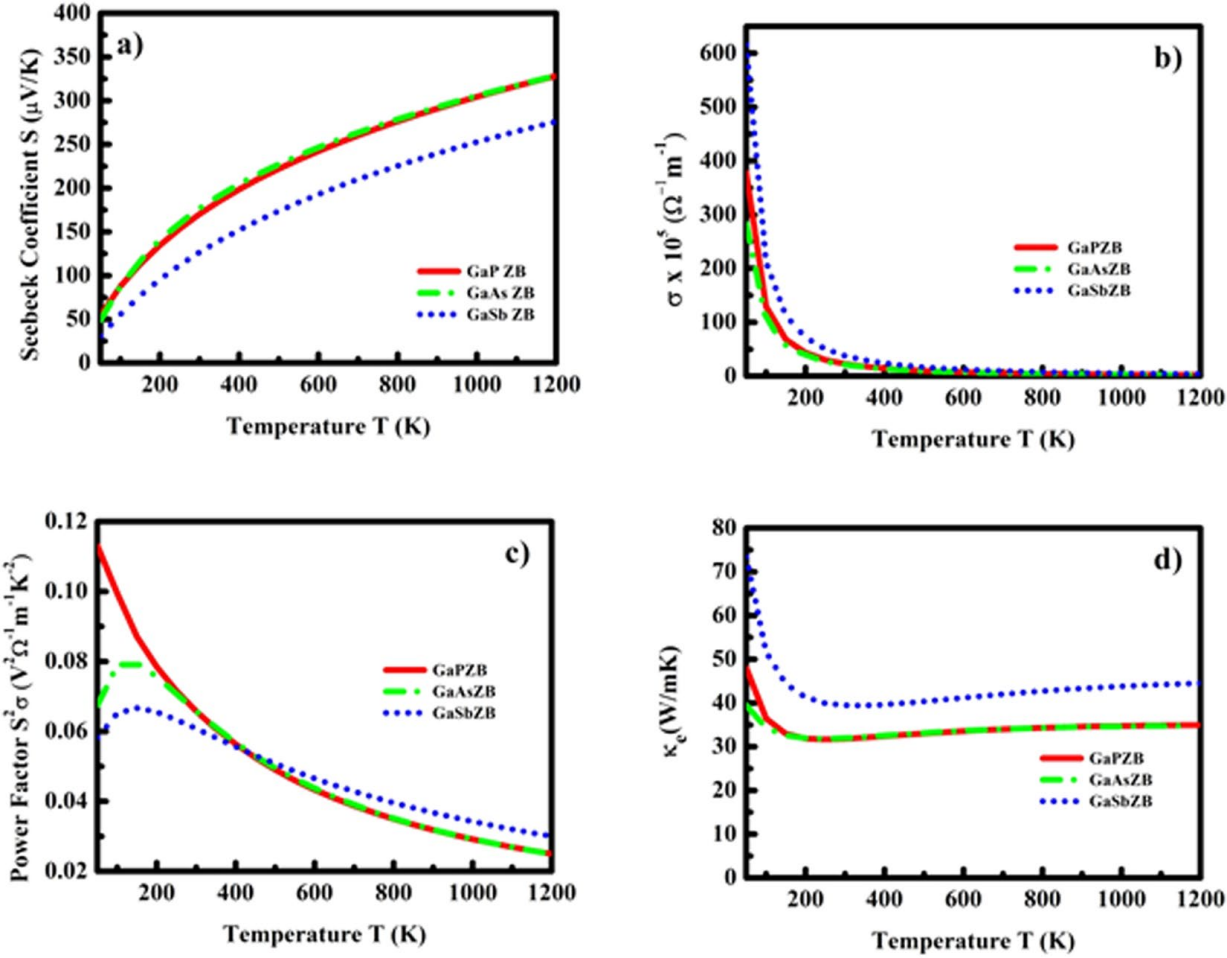
Thermoelectric parameters: (**a**) Seebeck co-efficient (S), (**b**) electrical conductivity (σ), (**c**) Power factor (S^2^σ) and (**d**) electronic conductivity (κ_e_) as a function of temperature for GaX compounds in ZB phase.

**Figure 9:**
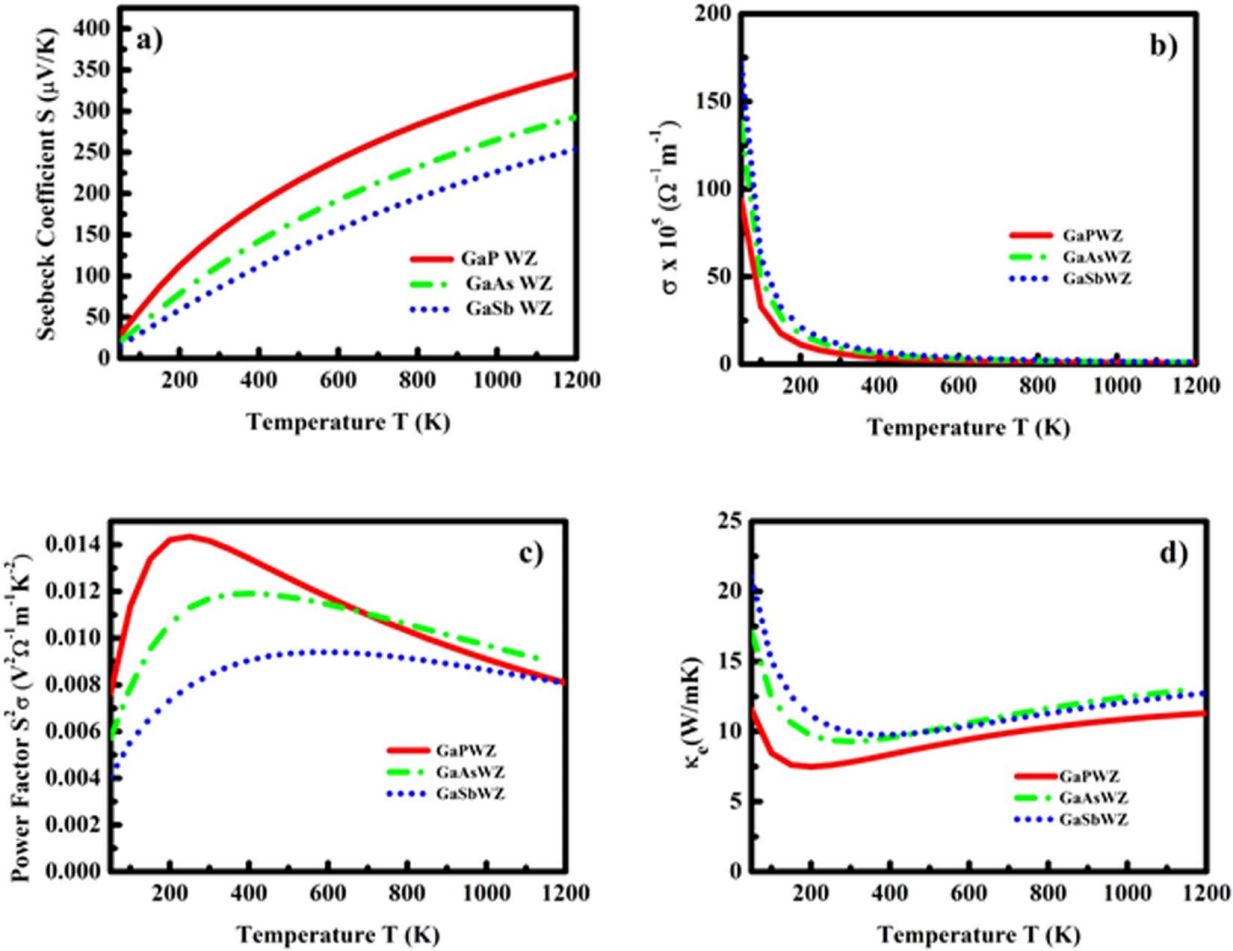
Thermoelectric parameters: (**a**) Seebeck co-efficient (S), (**b**) electrical conductivity (σ), (**c**) Power factor (S^2^σ) and (**d**) electronic conductivity (κ_e_) as a function of temperature for GaX compounds in WZ phase.

The thermoelectric parameters such as Seebeck co-efficient S, electric conductivity σ, power factor S^2^σ and electronic contributions to thermal conductivity κ_e_ as a function of temperature in the range 50 to 1200 K are presented in Figs 8 and 9 for ZB and WZ phases respectively. The nature of these quantities strongly depends on the carrier concentration “n”. Therefore, we first computed the power factor S^2^σ as a function of hole concentration “n” and observed a peak value of power factor at concentration 10^20^ cm^−3^. This hole concentration was then kept fixed for all systems and the remaining thermoelectric properties were computed as a function of temperature. As shown in Figs 8(a) and 9(a), the computed room temperature Seebeck co-efficient of GaP, GaAs and GaSbin ZB phase is about 170, 176 and 126 μV/K. It can be clearly observed that our computed value of Seebeck co-efficient for ZB GaAs is in excellent agreement with the prior experimental data (~170 μV/K)^66^, which validates our calculation and supports our utilized approach, although we could not compare our results of GaP and GaSb with any experimental data due to unavailability at same ambient conditions and carrier concentration. As observed from the Figs 8(a) and 9(a), the Seebeck co-efficient for all three compounds increases linearly with temperature and attains a maximum value about 328 and 345 μV/K at 1200 K for GaP in ZB and WZ phase respectively. The electrical conductivity σ, (see Figs 8(b) and 9(b)) show significant difference in magnitudes with similar trend in both phases. It can be observed that the σ (see Fig. 8(b)) decreases exponentially with increase in temperature. The temperature variation of power factor (S^2^σ) for ZB and WZ phases is shown in Figs 8(c) and 9(c) respectively. These figures reveal that the trend and magnitude of power factor are not same for both the phases. The room temperature values of power factor of GaP and GaAs in ZB phase is almost same (~0.065 V^2^Ω^−1^m^−1^K^−2^) while it is slightly lower (~0.060 V^2^Ω^−1^m^−1^K^−2^) for GaSb. The Fig. 9(c) shows the trend of power factor as a function of temperature of GaX compounds in WZ phase, and it is noteworthy, that the magnitudes of all three compounds show significant difference 0.014, 0.011 and 0.0084 V^2^Ω^−1^m^−1^K^−2^ at 300 K for GaP, GaAs and GaSb respectively. The electronic thermal conductivity κ_e_(see Figs 8(d) and 9(d)) shows reduction in low temperature regime (<200 K), after which it shows feeble enhancement with temperature and at 1200 K it attains the highest magnitude of 34.95, 34.78 and 44.52 W/mK and 11.32, 13.09 and 12.74 W/mK for GaP, GaAs and GaSb in ZB and WZ phases respectively. The enhanced values of power factors and lower values of electronic conductivity κ_e_ indicate the possibility of enhanced figure of merit ZT of these compounds if the thermal conductivity κ_l_ is also low.

**Figure 10:**
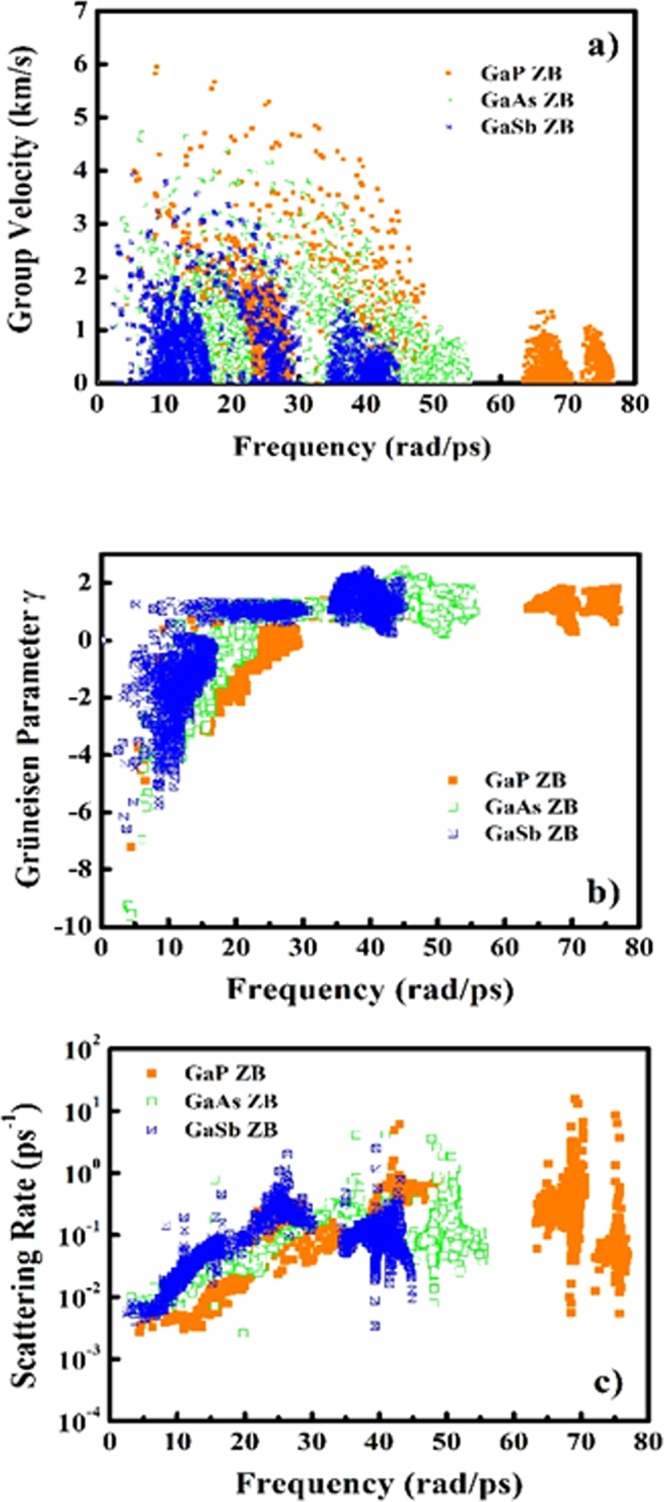
Phonon contribution to thermoelectric parameters: (**a**) Group velocity, (**b**) Grüneisen parameters (γ) and (**c**) Scattering rate as a function of vibrational frequency for GaX compounds in ZB phase.

**Figure 11:**
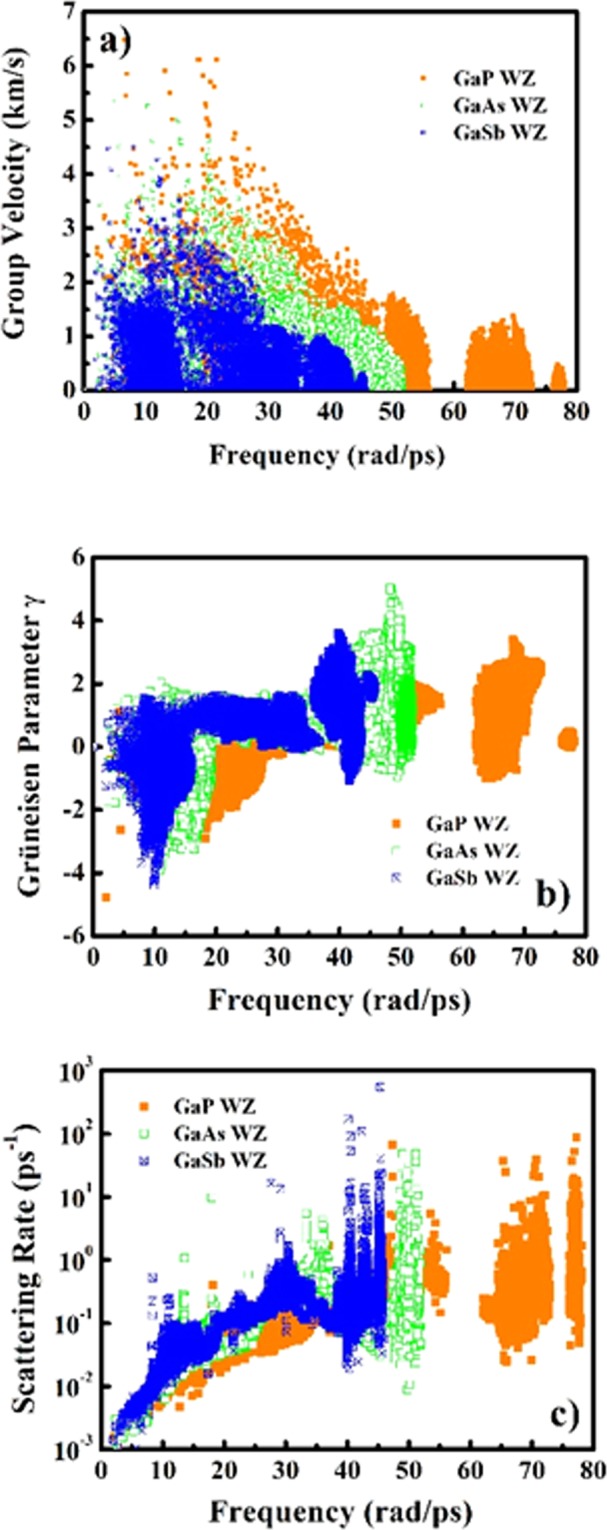
Phonon contribution to thermoelectric parameters: (**a**) Group velocity, (**b**) Grüneisen parameters (γ) and (**c**) Scattering rate as a function of vibrational frequency for GaX compounds in WZ phase.

After analyzing the electronic contributions to thermoelectric properties, we now turn our attention towards the underlying thermoelectric mechanism due to phonon contributions. For computing the phonon contribution to thermoelectric transport, the second order interatomic harmonic force constants (IFCs) generated under density functional perturbation approach (DFPT) approach^60^ which are utilized for further generating the third order interatomic and harmonic force constants (IFCs). The phonon dependent thermoelectric parameters such as group velocity, mode Grüneisen parameters and scattering rate as a function of frequency are presented in Figs 10 and 11. As it can be observed from Figs 10(a) and 11(a), the overall group velocity of the three acoustic modes attains higher magnitude for both phases of GaP with a dense accumulation in the case of WZ phase as the atomic contribution per unit cell is double than that of the ZB polytype. This trend of group velocity is also validated from the nature of acoustic phonon modes in PDCs (see Fig. 5). The acoustic phonon branches are steeper in the case of ZB phase than that of WZ phase. As a consequence of this, the large deviation in frequency with respect to wave vector **q** is observed which is the measure of the group velocity. The volume dependent mode Grüneisen parameter (γ) which is the measure of anharmonicity arising due to phonon-phonon scattering mechanism is shown in Figs 10(b) and 11(b) for ZB and WZ phases respectively. The WZ phase attains higher magnitude of γ than the ZB phase revealing stronger phonon anharmonicity and reduced phonon lifetimes. The trend of reduction in phonon lifetimes can be observed explicitly from the scattering rates of GaX compounds. Figures 10(c) and 11(c) present the phonon scattering rates as a function of phonon frequency. These plots reveal that the phonon-phonon scattering rates are higher for all WZ polytypes, and is highest observed for WZ GaP subjected to higher anisotropy between anion and cation masses. The comparison of the computed lattice thermal conductivity at 300 K with prior reported experimental and theoretical data is presented in Table 6.

**Table 6:** Lattice thermal conductivity (κ_l_) at 300 K for GaX compounds in ZB and WZ phases.

System	Phase	Lattice Thermal Conductivity κ_l_ (Wm^−1^K^−1^) at 300 K				
		Present	Theory^a^	Theory^b^	Theory^c^	Exp^d^
GaP	ZB	83.87	77	123	72	100
	WZ	39.96	—	—	—	—
GaAs	ZB	39.91	45	52	55	45
	WZ	31.1	—	—	—	—
GaSb	ZB	22.75	36	42	33	40
	WZ	18.58	—	—	—	—

^a^Ref.^53^ [Model].,

^b^Ref.^51^ [LDA]. and

^c,d^Ref.^68^ [Model + Exp].

**Figure 12:**
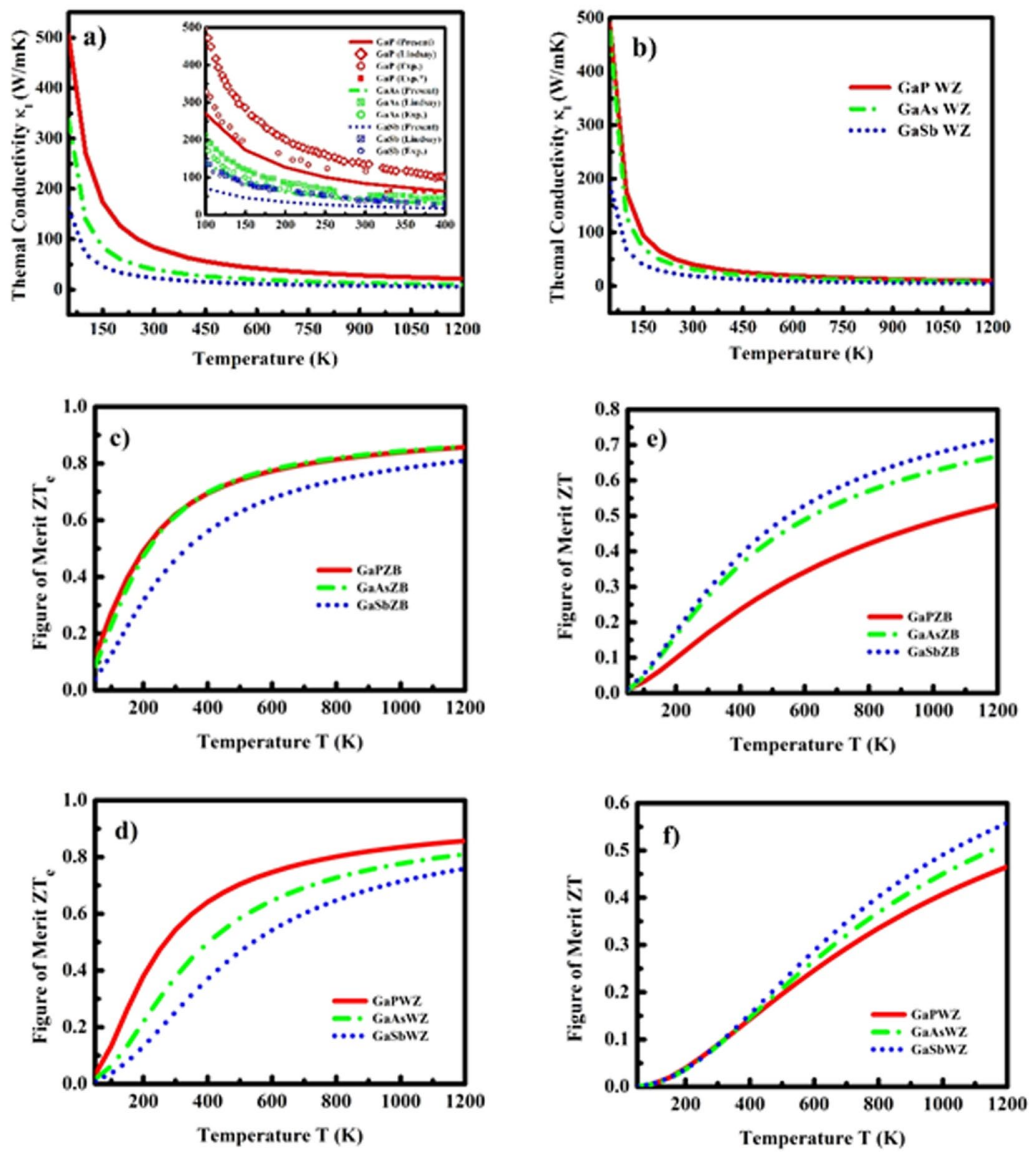
Thermal conductivity (κ_l_) of GaX compounds in ZB (**a**) (inset: comparison of (κ_l_) with available experimental and theoretical data) and (**b**) WZ phases, (**c**,**d**) electronic figure of merit (ZT_e_) and (**e**,**f**) overall figure of merit (ZT)as a function of temperature for ZB and WZ phase.

It can be observed that the computed lattice part of thermal conductivities are in reasonable agreement with the prior reports^53,67–71^, except with one of the DFT based report^51^ in which the isotope effect on thermal conductivity is considered. Moreover, it should be noted that the authors have performed the LDA based calculation for ZB compounds with inclusion of *d-orbital* electrons which might significantly affect the compound properties^61^. The dramatic difference in magnitude of lattice conductivity as a function of temperature of GaX compounds due to phase variance can be clearly observed in Fig. 12(a) and (b). The magnitude of κ_l_ is almost double for ZB GaP than WZ GaP pointing to the lower thermoelectric compatibility of ZB GaP; whereas for GaAs and GaSb, the difference in κ_l_ in both phases is comparable. Further, the inset of Fig. 12(a) represents the comparison of the present calculation and previously reported experimental and theoretical trends of thermal conductivity of GaX compounds in ZB phase. It is noteworthy that our computed values of thermal conductivity of GaX compounds show good agreement with the prior reports for all GaX compounds in ZB phase; however, the slight difference in the magnitudes can be attributed to the isotope effects which were not included in the present calculation. The low temperature trends (<300 K) of thermal conductivity reveal that the GaSb compound among the remaining two GaX compounds shows tremendous difference in the magnitude. We observe similar trend for WZ GaX compounds with crucial decrease in thermal conductivity especially observed for WZ GaSb (see Fig. 12(b)); these results predict signature contribution of WZ compounds in thermal management.

After computation of phonon contribution to thermoelectric properties, we assessed thermoelectric performance of these systems by computing the electronic and overall figure of merits ZT_e_ and ZT. The variation in ZT_e_ and ZT as a function of temperature is presented in Fig. 12(c) and (d) and Fig. 12(e) and (f) for ZB and WZ phases respectively. The trend of ZT_e_ is similar for all ZB and WZ compounds (see Fig. 12(c) and (d)); whereas in the case of magnitude, the GaP compound at 300 K possesses magnitude (~0.62 and 0.54) higher than GaAs(~0.61 and 0.37) and GaSb(~0.46 and 0.25) in ZB and WZ phases respectively. The ZT_e_ for both the phases show increase in magnitude with increase in temperature and after 800 K it almost show minor increase. Finally, the overall figure of merit ZT as a function of temperature which is shown in Fig. 12(e) and (f) shows a linear increase with temperature for both phases. In addition, Fig. 12(e) and (f) clearly show that the ZT is maximum for GaSb in both phases with magnitudes 0.29 and 0.088 at 300 K and, 0.72 and 0.56 at 1200 K in ZB and WZ phases respectively. Our results show that the overall values of ZT is higher for ZB phase. However, we expect that the nanowires of these compounds where the WZ phase is more probable, and may have higher ZT value. Among all GaX compounds, GaSb is claimed to be more suitable for thermoelectric applications as it possesses highest figure of merit ZT and lowest thermal conductivity κ_l_ with respect to other GaX compounds. Further, the higher value of ZT for all compounds in both phases at high temperature suggests that these compounds are suitable for high temperature thermoelectric devices.

## Conclusions

In summary, we have presented the theoretical results on structural, electronic, phonon and thermoelectric properties of three gallium pnictides (GaX) in ZB and WZ phases. This work employed the first principles pseudopotential electronic structure calculations in the DFT-LDA using norm conserving pseudopotentials. Our calculated results are in good agreement for structural and electronic parameters with the previously reported experimental and theoretical data with slight underestimation in lattice parameters. The band structure plots reveal the direct bandgap nature of GaAs and GaSb in both phases which indicate the importance of these compounds in optoelectronic applications. However, the bandgap for GaP in both phases is indirect. The PDCs show no imaginary components of vibrational frequency throughout the Brillouin zone validating the dynamic stability of GaX compounds in ZB and WZ phases at 0 GPa. The computed phonon frequencies are consistent with the reported experimental and theoretical data, except in the case of GaP in which the frequencies are slightly overestimated from experimental data. The thermodynamic functions computed under quasi harmonic approximation prove thermal stability of the system in both phases and supports for further analysis of the system for thermoelectric applications. Our thermoelectric computation of Seebeck co-efficient and relaxation time of GaAs in ZB phase validate the existing reports^65^ and hence give confidence in our approach. In a nut-shell, our results indicate GaSb compound to be more efficient for thermoelectric applications with overall figure of merit ZT, 0.72 and 0.56 in ZB and WZ phases respectively at 1200 K. In case of GaP and GaAs, the figure of merit is almost similar to GaSb in WZ phase and has dramatically lower magnitude for GaP than GaSb in ZB phase showing phase dependent anisotropy in thermoelectric transport. Our computed thermal conductivities of GaX compounds for ZB phase agree well with the experimental and previous results. However, our thermal conductivity results for WZ phase are predictive and could not be compared with either experimental or theoretical results. Further, the results on thermal conductivity also support GaSb to be more suitable for thermoelectric applications having lowest magnitude of lattice conductivity than other two GaX compounds with magnitudes 22.75 and 18.58 W/mK at 300 K in ZB and WZ phases respectively; however, these values are higher than the conventional materials used for thermoelectric applications. We attribute these higher magnitudes are subjected to the bulk phase of the compounds, which when subjected to confinement effect might affect the carrier effective masses and phonon mechanism resulting in enhancement in thermoelectric parameters. Further, the high-temperature (1200 K) thermal conductivities of GaP, GaAs and GaSb in ZB and WZ phases are 21.53, 9.99 and 5.8 W/mK and 9.53, 7.53 and 4.59 W/mK respectively, which strongly suggest GaSb to be most suitable for high-temperature thermoelectric applications than the remaining two GaX compounds. We also suggest that the GaP compoundis suggested to be less compatible at higher temperatures due to its highest thermal conductivity and lowest figure of merit among all three compounds.

## Methods

The *state-of the-art* first principles density functional theory (DFT) is utilized for investigating the ground state structural, electronic, vibrational, thermal and thermoelectric properties of gallium pnictides (GaX; X = P, As, Sb) in ZB and WZ phases^72^. The LDA treated exchange and correlation potential gives better prediction to ground state properties for III-V compounds over the GGA^55^ is utilized in which, the orbitals of Ga (4s^2^4p^1^), P (3s^2^3p^3^), As (4s^2^4p^3^), and Sb (5s^2^5p^3^) are treated as valence electrons for the total energy calculations. The exchange and correlation functionals were parameterized by employing the scheme proposed by Perdew and Zunger^57^ within scalar relativistic norm conserving pseudopotentials. The advantage of LDA pseudopotentials is that they are constructed on the assumption that the exchange and correlation energy of the inhomogeneous electron gas is the local analog to that of the corresponding homogeneous electron gas^73,74^. The optimization of the unit cell parameters like lattice constant, unit cell volume, unit cell angles, etc., of GaX compounds was done by minimizing the total energy of the systems using quasi-Newton Broyden-Fletcher-Goldfarb-Shanno algorithm. The total energy of the system was minimized self-consistently by fully relaxing the atomic positions with respect to cell shape, size and volume keeping the energy convergence value of 10^-4^ eV between two consecutive steps and, maximum Hellman-Feynman forces acting on each atom were less than 0.001 eV/Å to obtain well converged results. The high kinetic energy cut-off of 85 Rydberg (Ry) for the wave function was set for solving many body Kohn-Sham equation^72^. The Brillouin zone (BZ) integration along the high symmetry points was carried out with Monkhorst-Pack scheme based dense k-mesh grids of 16 × 16 × 16 and 16 × 16 × 10 for ZB and WZ phases respectively^75^. To understand the electronic transport through the GaX compounds in ZB and WZ phases, the electronic band structures and the density of states were computed along the respective high symmetry k-path of the first BZ. To confirm the dynamic stability of the structures and for further studying thermal and thermoelectric transport through the GaX system in ZB and WZ phases, the phonon dispersion curves (PDCs) with phonon density of states (PHDOS) were computed under density functional perturbation theory (DFPT) developed by Baroni *et al*.^60^ utilizing linear response approach. The inverse Fourier transformation to obtain phonon frequencies and corresponding eigenvectors is performed with compatible 6 × 6 × 6 and 6 × 6 × 4 **q**-mesh grids for ZB and WZ phases respectively. The thermodynamic functions such as specific heat (C_ν_), Debye temperature (Θ_D_), entropy (S), internal and vibrational energies of GaX compounds are computed under quasi-harmonic approximation (QHA). The electronic contribution to thermoelectric properties is computed by solving semi-classical Boltzmann transport equation utilizing BoltzTraP code^64^ and the phonon contribution to the same is obtained by solving phonon Boltzmann transport equation (PBTE) considering the third order phonon scattering effects as implemented on ShengBTE^76^ code. The semi-classical Boltzmann transport equations were solved under constant relaxation time approximation (CRTA), in which the Seebeck co-efficient of the system is independent of the scattering rates^77^, but also has a limitation as it computes relaxation time τ dependent electrical conductivity σ and electronic thermal conductivity κ_e_. To compute the τ independent accurate values of σ and κ_e_, we have computed the relaxation time τ for all the systems using the deformation potential theory proposed by Bardeen and Shokley^78^ which first gives the carrier mobility μ(see Equation (7)) and then utilizing μ, one can estimate the value of relaxation time τ (see Equation (8)).

7 $${\rm{\mu }}=\frac{{(8{\rm{\pi }})}^{\frac{1}{2}}{\hslash }^{4}{{\rm{eC}}}_{{\rm{ii}}}}{{({{\rm{m}}}^{\ast })}^{\frac{5}{2}}{({{\rm{k}}}_{{\rm{B}}}{\rm{T}})}^{\frac{3}{2}}{{\rm{E}}}_{1}^{2}}$$

where, μ is carrier mobility, e is electronic charge, *ħ* is reduced Plank’s constant, C_ii_ represents the elastic constant of the system, k_B_ is Boltzmann co-efficient, T is temperature, m^*^ is the carrier effective mass and E_1_ represents the deformation potential constant. For computing the deformation potential E_1_, the unit cells were relaxed under the influence of external strain in the range of ±3% with 0.5% step size and the valence band maxima (VBM) energies of the structures were computed. The valence band maxima (E^VBM^) energies as well as the lowest energy eigen values (E^Core^) of the structures were computed under the influence of strain. Then we had aligned the E^VBM^ with respect to lowest energy level (E^Core^) by taking the difference between E^VBM^ and E^Core^. This aligned energy eigen values were plotted with respect to applied strain and then the deformation potential have been calculated using the formula^79^ *d*(E^VBM^ − E^Core^)/*dv*. Here, *v* is volume strain = ΔV/V_0_ with ΔV = V − V_0_; V and V_0_ being the volumes of unit cell under strained and equilibrium conditions respectively.

The aligned energy eigen values were plotted as a function of ΔV/V_0_ which was then fitted and the slope (δ(E^VBM^ − E^Core^)/δ(ΔV/V_0_)) was extracted, defined here as deformation potential constant E_1_. The relaxation time τ which is the time between two successive collisions of electron and ions is computed by using the relation given below.

8 $$\tau =\frac{{{\rm{\mu }}m}^{\ast }}{{\rm{e}}}$$

The optimization of the GaX compounds and computations of the proposed properties were performed using plane wave Quantum Espresso distribution^80^.

## Supplementary information

Supplementary Information
