# Supplementary material for: ab initio Energetics and Thermoelectric Profiles of Gallium Pnictide Polytypes
Source: Sci Rep. 2019 Apr 10;9:5884. doi: 10.1038/s41598-019-41982-9 (PMC6458143; doi:10.1038/s41598-019-41982-9)
Supplement: Supplementary file 1 — Supplementary Information [file 41598_2019_41982_MOESM1_ESM.docx]

Supplementary Information for:

***ab initio* Energetics and Thermoelectric Profiles of Gallium Pnictide Polytypes**

**Trupti K Gajaria^a^, Shweta D. Dabhi^b^ and Prafulla K. Jha^a,*^**

**^a^***Department of Physics, Faculty of Science, The Maharaja Sayajirao University of Baroda, Vadodara-390002, Gujarat, India.*

*^b^P. D. Patel Institute of Applied Science, Charotar University of Science and Technology, CHARUSAT campus, Changa-388421, Gujarat, India.*

[^*^prafullaj@yahoo.com](file:///C:\Users\Tejal.NOVA\Desktop\research\GaX(X=As,P,Sb)\Scientific%20Reports\*prafullaj@yahoo.com)

Supplementary Figure Captions

**Figure S1.** Power factor (S^2^σ) as a function of hole concentration (n) for GaX compounds in ZB and WZ phases.

**Figure S2.** The computed carrier mobility μ as a function of temperature for GaX compounds in ZB and WZ phases.

**Figure S3.** The computed relaxation time τ as a function of temperature for GaX compounds in ZB and WZ phases.


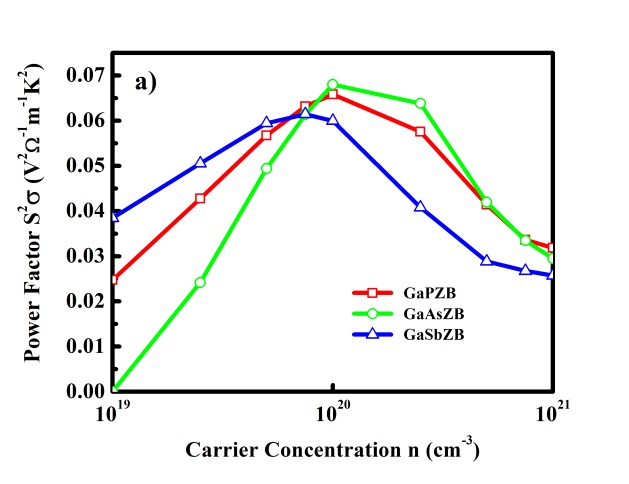

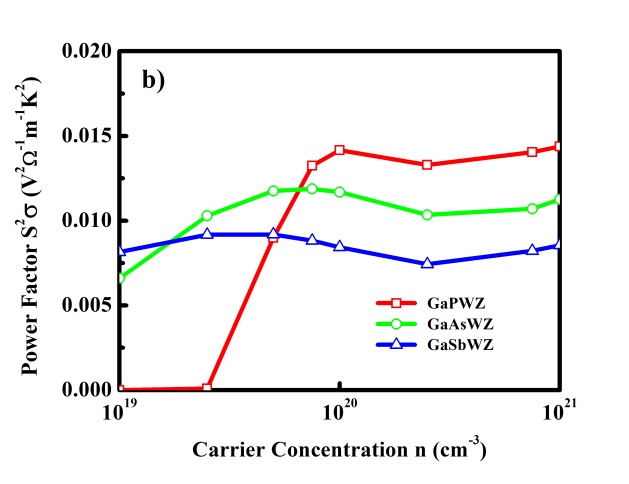


**Supplementary Figure S1**


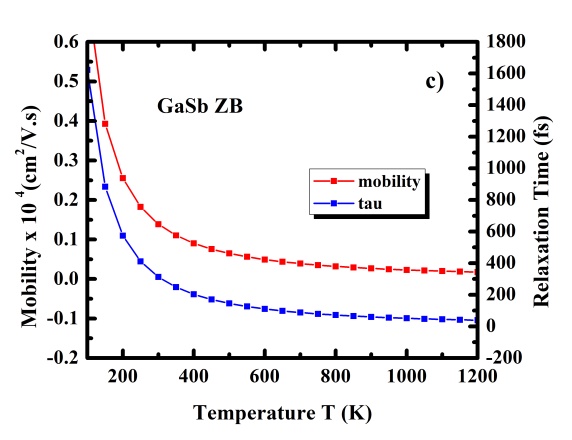

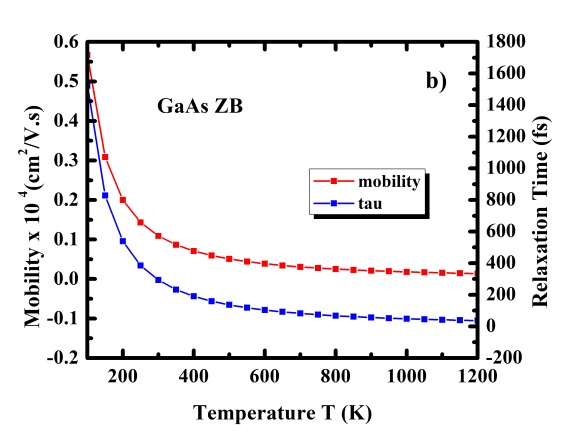

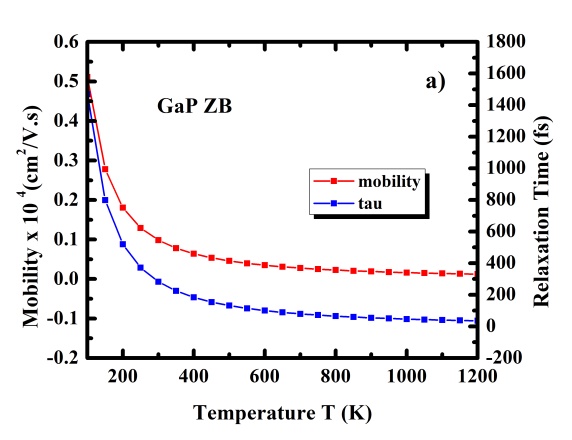


**Supplementary Figure S2**

**Supplementary Figure S3**


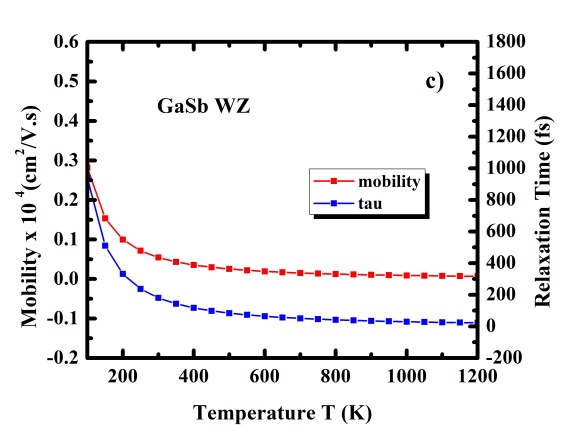

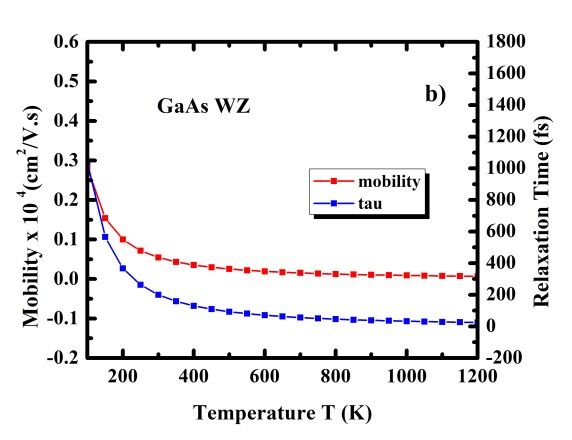

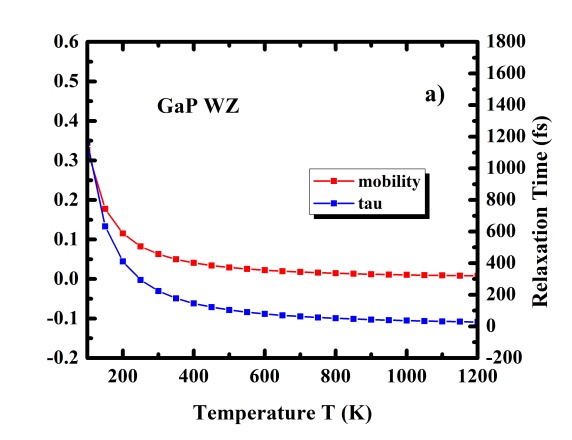


**Text for supplementary figures S1, S2 and S3**

**Supplementary Figure S1**

For computing the optimum magnitude of hole concentration, the power factor S^2^σ was evaluated for various hole concentrations (n) at 300 K temperature for GaX compounds in ZB and WZ phases and the peak value of power factor was observed at hole concentration 10^20^ cm^-3^. The hole concentration was then kept fixed at this value for all GaX compounds and the remaining thermoelectric parameters were computed as a function of temperature.

**Supplementary Fig S2 and S3**

Figures S2 and S3 (a-c) represent the temperature dependent hole mobility (µ) and hole relaxation time (τ) for GaX compounds in ZB and WZ phases respectively. The hole relaxation time was explicitly computed by calculating hole mobility under deformation potential approach proposed by Bardeen and Shokley (see Methods section of main article for more details). It can be observed from the figures S2 and S3 (a-c) that both µ and τ decrease exponentially with rise in temperature indicating higher rate of collision.
